# Microarray analysis of long non-coding RNA expression profiles in monocytic myeloid-derived suppressor cells in *Echinococcus granulosus*-infected mice

**DOI:** 10.1186/s13071-018-2905-6

**Published:** 2018-05-30

**Authors:** Aiping Yu, Ying Wang, Jianhai Yin, Jing Zhang, Shengkui Cao, Jianping Cao, Yujuan Shen

**Affiliations:** 1 National Institute of Parasitic Diseases, Chinese Center for Disease Control and Prevention, Shanghai, 200025 China; 2 Key Laboratory of Parasite and Vector Biology, MOH, Shanghai, 200025 China; 3 National Center for International Research on Tropical Diseases, Shanghai, 200025 China; 4 WHO Collaborating Centre for Tropical Diseases, Shanghai, 200025 China

**Keywords:** *Echinococcus granulosus*, Myeloid-derived suppressor cells, Long non-coding RNAs, Expression profile, Microarray analysis

## Abstract

**Background:**

Cystic echinococcosis is a worldwide chronic zoonotic disease caused by infection with the larval stage of *Echinococcus granulosus*. Previously, we found significant accumulation of myeloid-derived suppressor cells (MDSCs) in *E. granulosus* infection mouse models and that they play a key role in immunosuppressing T lymphocytes. Here, we compared the long non-coding RNA (lncRNA) and mRNA expression patterns between the splenic monocytic MDSCs (M-MDSCs) of *E. granulosus* protoscoleces-infected mice and normal mice using microarray analysis.

**Methods:**

LncRNA functions were predicted using Gene Ontology enrichment and the Kyoto Encyclopedia of Genes and Genomes pathway analysis. *Cis*- and *trans*-regulation analyses revealed potential relationships between the lncRNAs and their target genes or related transcription factors.

**Results:**

We found that 649 lncRNAs were differentially expressed (fold change ≥ 2, *P* < 0.05): 582 lncRNAs were upregulated and 67 lncRNAs were downregulated; respectively, 28 upregulated mRNAs and 1043 downregulated mRNAs were differentially expressed. The microarray data was validated by quantitative reverse transcription-PCR. The results indicated that mRNAs co-expressed with the lncRNAs are mainly involved in regulating the actin cytoskeleton, *Salmonella* infection, leishmaniasis, and the vascular endothelial growth factor (VEGF) signaling pathway. The lncRNA NONMMUT021591 was predicted to *cis*-regulate the retinoblastoma gene (*Rb1*), whose expression is associated with abnormal M-MDSCs differentiation. We found that 372 lncRNAs were predicted to interact with 60 transcription factors; among these, C/EBPβ (CCAAT/enhancer binding protein beta) was previously demonstrated to be a transcription factor of MDSCs.

**Conclusions:**

Our study identified dysregulated lncRNAs in the M-MDSCs of *E. granulosus* infection mouse models; they might be involved in M-MDSC-derived immunosuppression in related diseases.

**Electronic supplementary material:**

The online version of this article (10.1186/s13071-018-2905-6) contains supplementary material, which is available to authorized users.

## Background

Cystic echinococcosis is a worldwide chronic zoonotic disease caused by accidental ingestion of eggs of the genus *Echinococcus* and typically affects the liver and lungs [1]. It is endemic in pastoral regions around the world [2], causes a huge disease burden, and is characterized by long-term growth of hydatid cysts in humans and mammalian intermediate hosts. The cysts are filled with hydatid cyst fluid and protoscoleces [3].

Previously, we found significant accumulation of myeloid-derived suppressor cells (MDSCs) in mouse models infected with *E. granulosus* protoscoleces (Eg-psc) [4] and that they play a key role in downregulating the immune response of T lymphocytes. MDSCs are a heterogeneous population of myeloid cells composed of terminally differentiated macrophages, granulocytes, or dendritic cells. Various pathological conditions, such as cancer [5, 6], sepsis [7] and parasitic infection [8] result in aberrant MDSC expansion. MDSCs consist of two major subsets based on their phenotypic and morphological features: polymorphonuclear (PMN)-MDSCs and monocytic (M)-MDSCs. In mice, they are historically characterized by concurrent expression of the myeloid markers CD11b and Gr-1. The two major subsets can be identified more accurately based on the expression of Ly6G and Ly6C markers: PMN-MDSCs, CD11b^+^Ly6G^+^Ly6C^low^; M-MDSCs, CD11b^+^Ly6G^-^Ly6C^hi^) [9, 10]. M-MDSCs and PMN-MDSCs inhibit immune function *via* different mechanisms. M-MDSCs suppress T cell function *via* both antigen-specific and nonspecific mechanisms by producing nitric oxide (NO) and cytokines [11, 12], and are more immunosuppressive than their counterparts when assessed on a per cell basis [13–15].

Long non-coding RNAs (lncRNAs) are commonly defined as transcribed RNAs of more than 200 nucleotides in length and lack protein-coding ability [16, 17]. Increasing evidence indicates that lncRNAs participate in several important biological processes, including carcinogenesis, cell differentiation, metabolism, and immunity responses [18–20], acting as signal molecules, decoys, guides, and scaffolds [21–23]. Although numerous lncRNAs have been discovered in recent years, only a limited number have been well characterized. At the same time, knowledge of the genome scale of lncRNAs and their underlying biological functions in MDSCs in parasitic infections remains limited. Moreover, MDSC functional plasticity *via* epigenetic modification leads to their characteristics reshaping [10].

In the present study, we used microarray analysis to investigate the lncRNA and mRNA expression profiles in the splenic M-MDSCs of normal and Eg-psc-infected mice, and performed bioinformatics analysis of the differentially expressed lncRNAs to explore the possible biological processes and pathways associated with M-MDSCs. The results demonstrate that aberrantly expressed lncRNAs may be new candidates for the immunosuppressive mechanism of M-MDSCs in parasitic diseases.

## Methods

### Mice, parasites, and infection

Female BALB/c mice (aged 6–8 weeks) were purchased from SLAC Laboratory. The Eg-psc were obtained from the hydatid cysts of naturally infected sheep livers under aseptic conditions, and washed three times using 0.9% NaCl containing 1000 mg/ml penicillin and 1000 U/ml streptomycin (Invitrogen, Frederick, MD, USA). Thirty BALB/c mice were intraperitoneally injected with a 200 μl suspension containing 2000 live Eg-psc in 0.9% NaCl; the controls were 30 BALB/c mice injected with 200 μl 0.9% NaCl. All mice were housed in specific pathogen-free conditions.

### Cell isolation

Splenic M-MDSCs were isolated immediately after the BALB/c mice were sacrificed under sterile conditions at eight months after infection. Single-cell suspensions were enriched with magnetic cell sorting (MACS; Miltenyi Biotec, Bergisch Gladbach, Germany) according to the manufacturer’s protocol. The M-MDSCs were separated on mini MACS columns (Miltenyi Biotec, Bergisch Gladbach, Germany) and yielded approximately 90% pure cells. Then, we randomly selected splenic M-MDSCs from three mice each in the infected and normal groups for the detection of lncRNA and mRNA arrays.

### Microarray profiling

The lncRNA and mRNA expression patterns were detected in the splenic M-MDSCs of three Eg-psc-infected mice and three normal mice. The experiments were performed at OE BioTechCorporation (Shanghai, China). Agilent mouse lncRNA Microarray (4*180K, Design ID: 049801) was used in this experiment. Total RNA was extracted and purified with a RNeasy Mini Kit (Qiagen, p/n 74104, Boston, MA, USA) and quantified using NanoDrop ND-2000 (Thermo Fisher Scientific, Waltham, MA, USA). RNA integrity was assessed using an Agilent Bioanalyzer 2100 (Agilent Technologies, Santa Clara, CA, USA). Sample labeling, microarray hybridization, and washing were performed based on the manufacturer’s standard protocols. Briefly, total RNA was transcribed to double-stranded complementary DNA (cDNA), synthesized into complementary RNA (cRNA), and labeled with Cyanine 3-CTP. The labeled cRNA was hybridized onto the microarray. After washing, the arrays were scanned using an Agilent Scanner G2505C microarray scanner (Agilent Technologies).

### Differential expression analysis

The raw data were analyzed using Feature Extraction software (version 10.7.1.1; Agilent Technologies) and then normalized using percentile normalization. Probes with least one of two conditions flagged in “P” were chosen for further data analysis. Differentially expressed lncRNAs were identified through fold change and the *P*-value as calculated with the t-test. Aberrantly expressed lncRNAs and mRNAs were defined as fold change ≥ 2.0 and *P* < 0.05. Subsequently, Gene Ontology (GO) enrichment and Kyoto Encyclopedia of Genes and Genomes (KEGG) analyses were used to explore the roles of the differentially expressed mRNAs. Hierarchical clustering was performed on six mouse splenic tissue samples using Cluster 3.0 (Stanford University School of Medicine, California, USA) and TreeView 2.0 (Baryshnikova Lab, Princeton University, New Jersey, USA) to distinguish the distinguishable gene expression pattern among the samples.

### Co-expression network analysis

The co-expression of lncRNAs and the protein-coding genes was calculated using Pearson correlation coefficients with Cytoscape version 3.1.1 (US National Institute of General Medical Sciences). Correlations with *P* < 0.05 were considered statistically significant.

### Functional enrichment analysis

The functions of lncRNA co-expressed mRNAs were analyzed using GO enrichment analysis which was divided into three functional categories: molecular function, biological process and cellular component. The pathways of the co-expressed mRNAs were analyzed using KEGG pathway analysis.

### Quantitative reverse transcription-PCR (qRT-PCR)

qRT-PCR was performed to validate the microarray results according to the manufacturer’s protocol with a SYBR Green RT-PCR Kit (QuantiFast SYBR Green PCR Master Mix, Qiagen) on a Bio-Rad CFX96 system. The primers used for the qRT-PCR are shown in Additional file 1: Table S1. The lncRNA expression levels were quantified based on the threshold cycle (Ct) values. β-Actin served as the internal control. The relative gene expression was analyzed using the comparative Ct [2(-ΔΔCt)] method [24]. Three biological replicates were performed for each group.

### *Cis*- and *trans*-regulation analysis

LncRNAs have been shown to enhance the expression of nearby genes through *cis*-regulation [25] and as the mRNA loci were within 300 kbp windows upstream and downstream of the given lncRNAs, we identified them as *cis*-regulated mRNAs of the corresponding lncRNAs.

Here, the lncRNAs identified showed high Pearson’s correlation with the neighboring protein-coding genes, suggesting that they act in *cis* on protein-coding genes to regulate M-MDSC functions. As it has been indicated that transcription factors regulate lncRNA production, we therefore used hypergeometric distribution testing to predict the potential transcription factors that may regulate the production of the differentially expressed lncRNAs.

### Statistical analysis

Statistical analysis was performed using SPSS 19.0 (SPSS, New York, USA). All measurement data are reported as the mean ± standard deviation (SD). Differentially expressed lncRNAs were identified using t-tests or nonparametric tests. *P* < 0.05 was considered statistically significant.

## Results

### Differentially expressed lncRNAs in M-MDSCs

The raw data were analyzed using Feature Extraction software (version 10.7.1.1, Agilent Technologies) and were normalized with the quantile algorithm. GeneSpring (version 13.1, Agilent Technologies) was used for basic raw data analysis. Probes with at least 100% of the values in any one out of all conditions flagged as “Detected” were selected for further analysis. Differentially expressed mRNAs or lncRNAs were identified through fold change and the *P*-value as calculated with the t-test. The threshold set for upregulated or downregulated RNAs was fold change ≥ 2.0 and *P* < 0.05.

We detected 54,030 lncRNAs and 33,420 mRNAs in the M-MDSC samples. Microarray scanning and normalization determined that 649 lncRNAs and 1071 mRNAs were differentially expressed. Among them, 582 lncRNAs were upregulated and 67 lncRNAs were downregulated (Additional file 2: Table S2), and 28 upregulated and 1043 downregulated mRNAs were differentially expressed (Additional file 3: Table S3). Compared with the controls, FR208893 (fold change of 133.45992) was the most upregulated lncRNA, while FR325025 (fold change of 4.1526523) was the most downregulated lncRNA in the M-MDSCs.

**Fig. 1:**
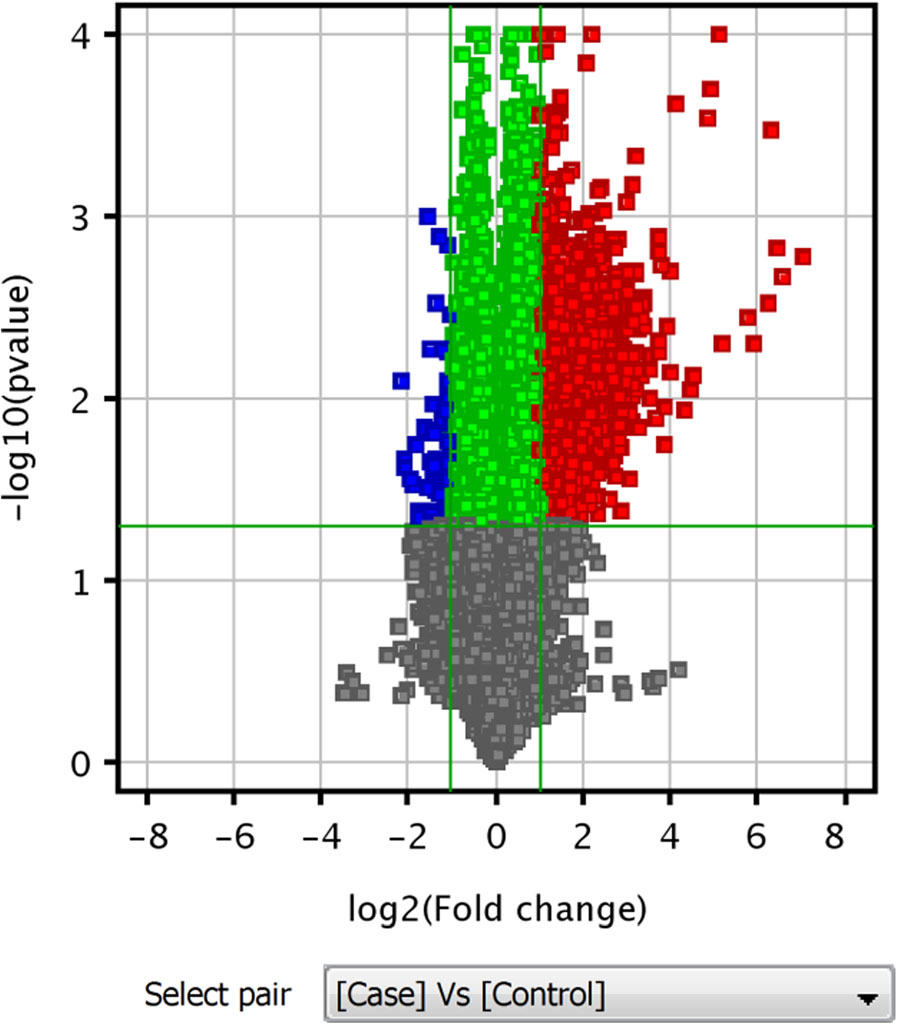
Aberrant expression of lncRNAs between the two groups. The X-axis indicates the fold change. The horizontal green line represents the filter criterion (threshold *P* ≥ 0.05); red dots, upregulated lncRNAs; blue dots, downregulated lncRNAs

**Fig. 2:**
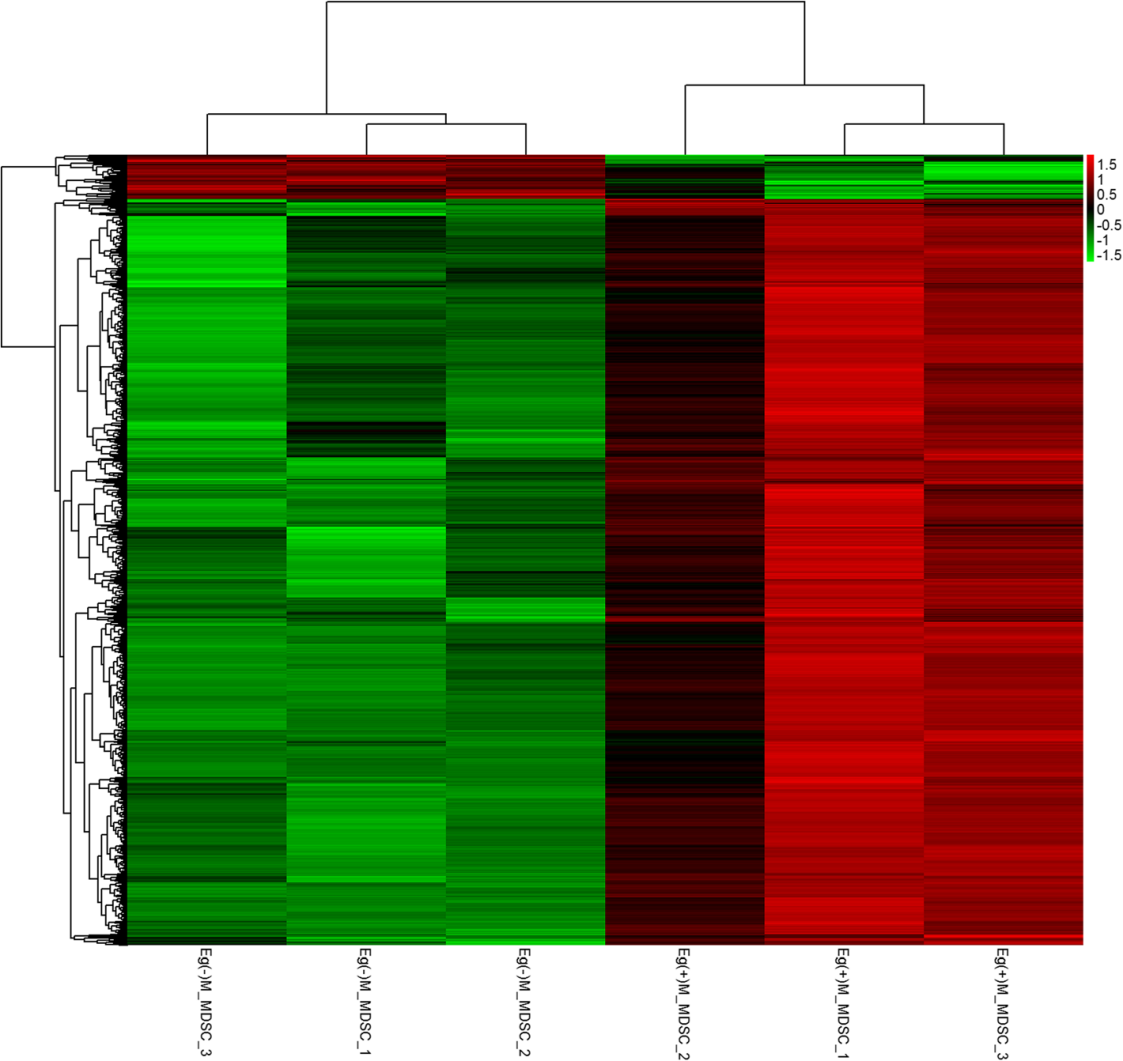
Hierarchical clustering of differentially expressed lncRNAs between the splenic M-MDSCs of normal and Eg-psc-infected mice. In the heat map, red indicates increased relative expression and green indicates decreased relative expression

Figure 1 shows a volcano plot of the differentially expressed lncRNAs, where red and green indicate significantly upregulated and downregulated lncRNAs, respectively. To identify gene expression patterns, hierarchical clustering was performed among samples using Cluster 3.0 and TreeView 2.0. Many lncRNAs were identified as differentially expressed between the two groups, indicating that the significantly altered expression of these lncRNAs may be involved in the immunoregulatory function of M-MDSCs (Fig. 2).

### qRT-PCR validation

**Fig. 3:**
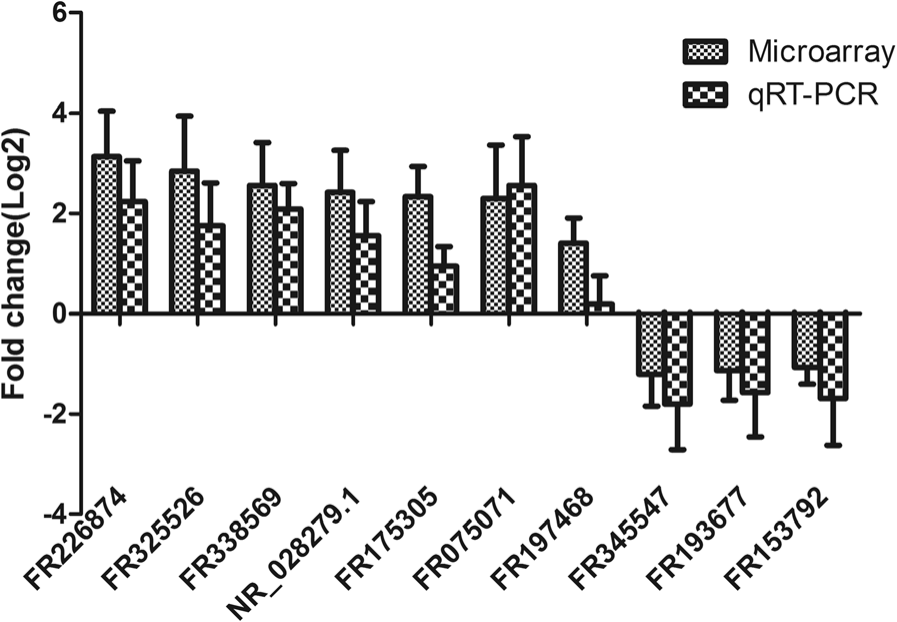
qRT-PCR verification of 10 randomly selected differentially expressed lncRNAs from the microarray data. The qRT-PCR results were consistent with the lncRNA array analysis. Results are presented as log2 fold changes in expression ± standard error

To verify the results of lncRNA microarray, we used qRT-PCR to detect the expression of 10 lncRNAs selected randomly from the differentially expressed lncRNA transcripts with splenic M-MDSCs samples with three biological replicates in each group. The qRT-PCR results were consistent with the lncRNA array analysis, and these lncRNAs are likely to play roles in response to the biological functions of MDSCs in parasitic infections (Fig. 3).

### LncRNAs-mRNAs co-expression network

We constructed a co-expression network to investigate the correlation between each differentially expressed lncRNA in M-MDSCs and the target mRNAs. We ranked each lncRNA-mRNA correlation according to the *P*-value and selected the top 500 lncRNA functional terms for functional enrichment analysis, and counted the numbers of differently expressed lncRNAs enriched in the functional terms. These functional terms were used to predict the functions of the given lncRNAs based on GO enrichment and KEGG pathway analyses of the co-expressed mRNAs.

**Fig. 4:**
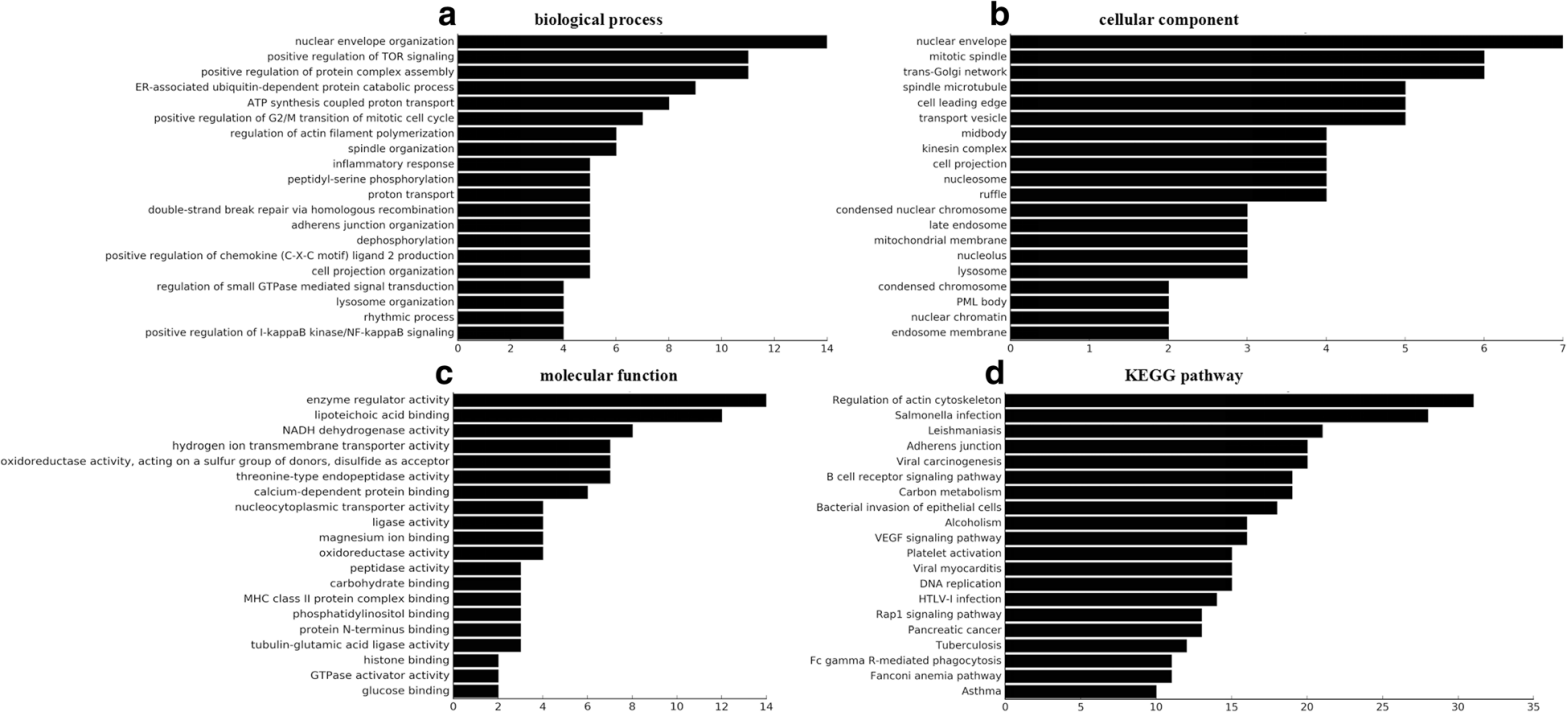
Top 20 terms in the gene enrichment and pathway analysis of differentially expressed lncRNAs induced by Eg-psc infection. **a** Biological process. **b** Cellular component. **c** Molecular function. **d** The most significant KEGG pathway for the differentially expressed lncRNAs

For GO analysis, differentially expressed lncRNAs were mostly enriched in nuclear envelope organization, positive regulation of target of rapamycin (TOR) signaling and positive regulation of protein complex assembly in biological processes (Fig. 4a); nuclear envelope, mitotic spindle, and trans-Golgi network in cellular component (Fig. 4b), and enzyme regulator activity, lipoteichoic acid binding, and NADH dehydrogenase activity in molecular functions (Fig. 4c).

It is widely believed that disorder of the signaling pathways under pathological conditions contributes to the development of suppressive myeloid cells [26]. KEGG pathway analysis indicated that the co-expressed mRNAs were mainly involved in regulating the actin cytoskeleton, *Salmonella* infection, leishmaniasis, and the vascular endothelial growth factor (VEGF) signaling pathway (Fig. 4d). MDSCs accumulate in lymphoid organs under parasitic infection (as in cancer), and can migrate to and invade the adjacent tissues and vasculature. Some key proteins involved in the actin cytoskeleton are linked to cancer cell invasion and metabolism [27]. As cystic echinococcosis is considered an infectious and inflammatory disease, the aberrantly expressed lncRNAs might regulate these processes.

### *Cis*-regulation of lncRNAs

We found that 288 lncRNAs were considered *cis*-regulatory lncRNAs of their sense-overlapping genes, and we found that lncRNA NONMMUT021591 was predicted to *cis*-regulate the *Rb1* (Additional file 4: Figure S1). *Rb1* expression had been reported to be associated with abnormal M-MDSC differentiation [28].The lncRNA NONMMUT021591 may play a role in the immunosuppressive functions of MDSCs.

### LncRNA-transcription factor network analysis

**Fig. 5:**
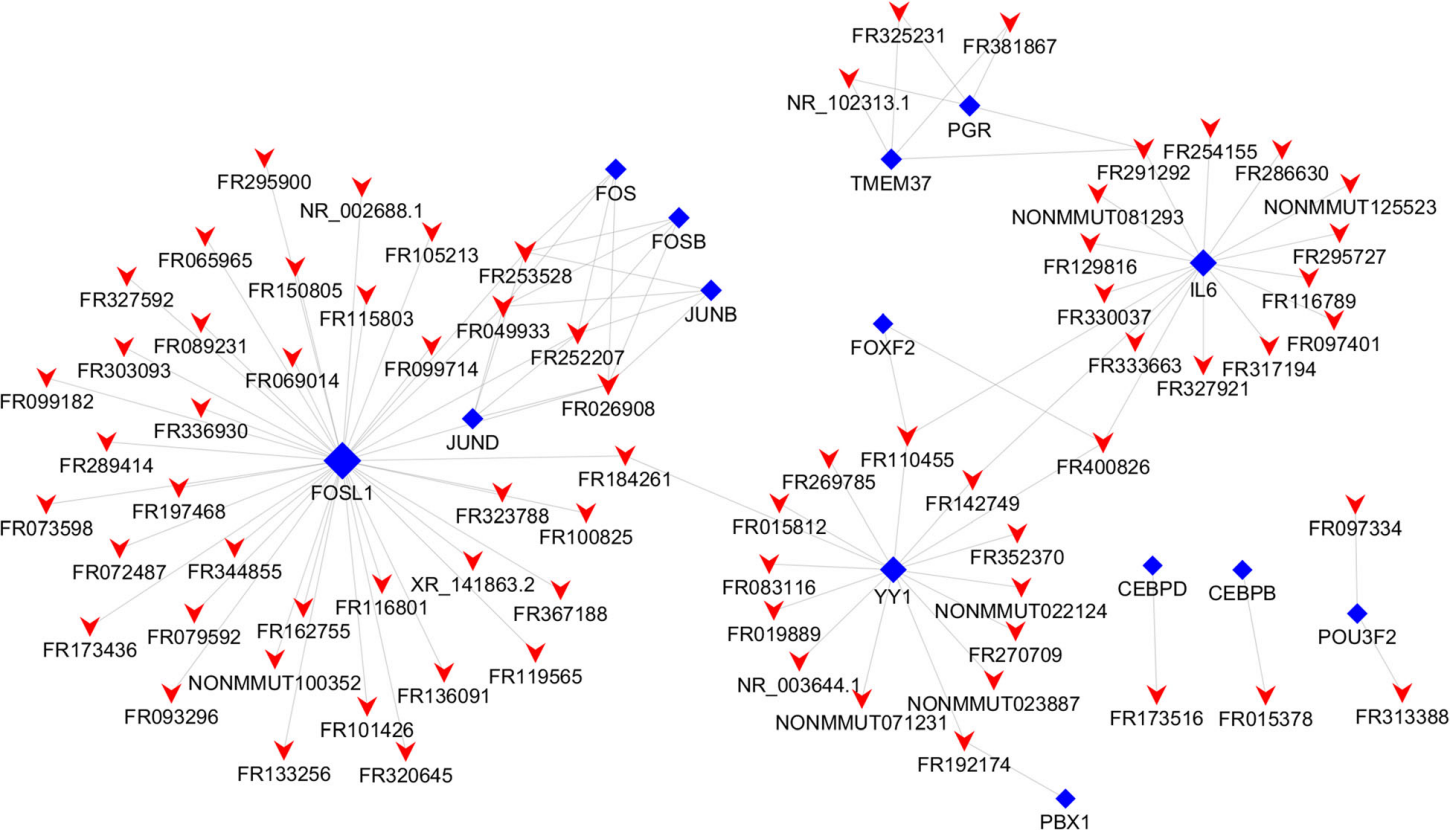
Network of the top 100 most related lncRNA-transcription factor pairs according to the *P*-value. Red arrowheads, lncRNAs; blue diamonds, transcription factors

As it was indicated that transcription factors regulate lncRNA production, we used hypergeometric distribution testing to predict the transcription factors that could regulate the differentially expressed lncRNAs, and constructed a core network of the top 100 lncRNA-transcription factor pairs by ranking the *P*-value (Fig. 5). We predicted that 372 lncRNAs would interact with 60 transcription factors, forming an interaction network of 1746 connections. These lncRNAs were mostly regulated by IL6, FOSL1, YY1, PGR, TMEM37, PBX1, POU3F2, FOXF2, FOS, JUNB, JUND, CEBPD, RB1, MAX, E2F1, C/EBPβ, HERPUD1, E2F4 and ZBTB16. Among the transcription factors, C/EBPβ (CCAAT enhancer-binding transcription factor) [29] has been demonstrated to be a MDSC transcription factor. C/EBPβ regulates myeloid cell development and differentiation, and controls emergency granulopoiesis induced by cytokines and infections [7, 30]. Increased C/EBPβ expression is a characteristic biochemical feature of MDSCs.

### LncRNA-target-transcription factor network analysis

**Fig. 6:**
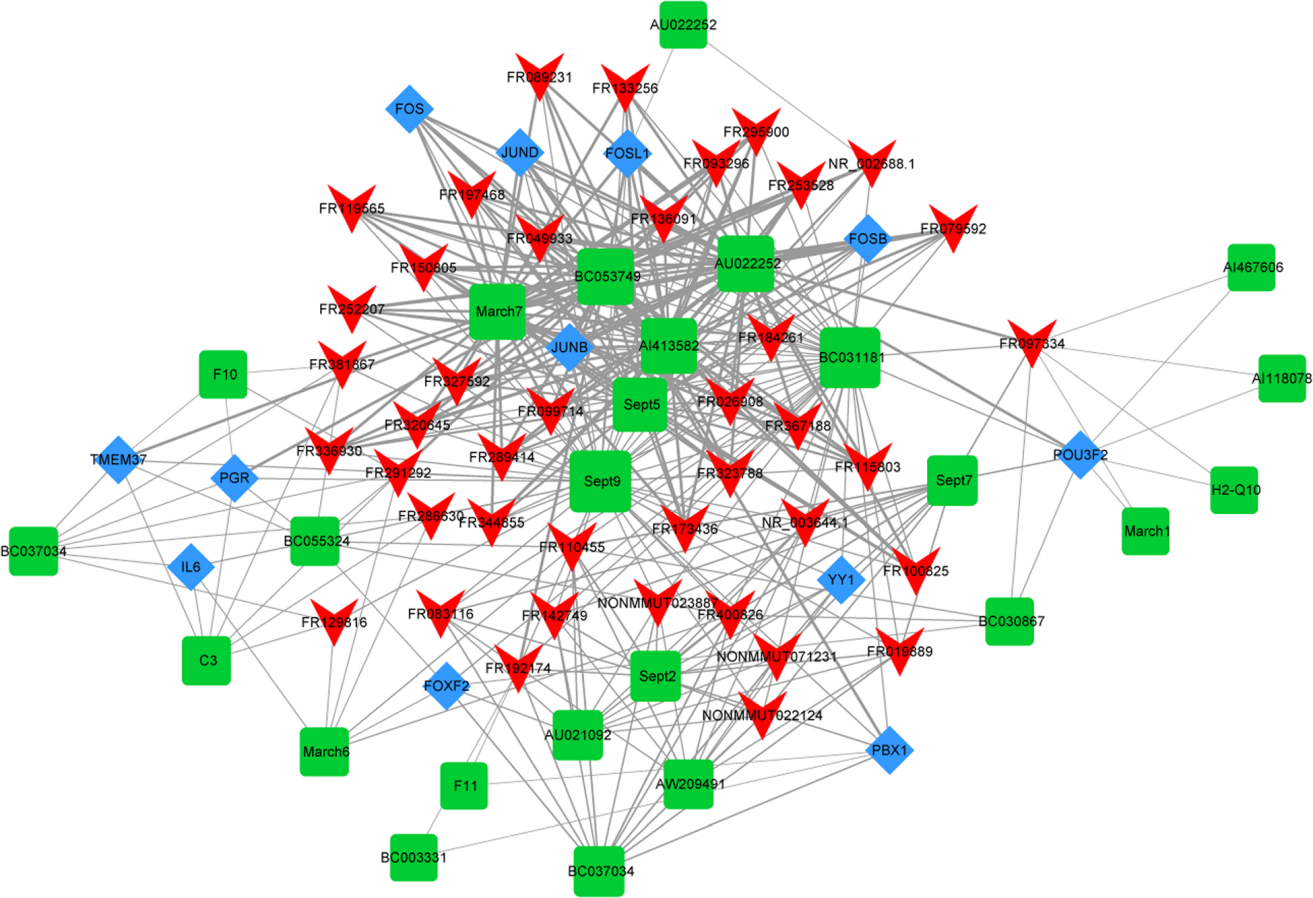
Network of the top 50 most related lncRNA-target-transcription factors. Red arrowheads, lncRNAs; green diamonds, mRNAs; blue diamonds, transcription factors

For further identification of the functions of each dysregulated lncRNA in M-MDSCs, we analyzed the top 50 differentially expressed lncRNAs and their co-expressed mRNAs pairs according to the *P*-value to conduct the lncRNA-target-transcription factor network (Fig. 6). The network revealed several most likely transcription factors for these lncRNAs, and included FOSL1, YY1, IL6 and PGR. LncRNAs FR049933, FR291292, FR110455 and FR400826 are predicted to be mainly regulated by these transcription factors and participate in mitogen-activated protein kinase (MAPK) signaling pathway and VEGF signaling pathway, which are involved in MDSC function.

## Discussion

Cystic echinococcosis is a worldwide chronic zoonotic disease in pastoral regions around the world [2]. Transmission is through predator-prey interactions between carnivore definitive hosts and herbivore intermediate hosts [31, 32]. Previously, we showed significant accumulation of CD11b^+^Gr-1^+^ myeloid cells in the spleen and peripheral blood in Eg-psc-infected mouse models [4] . Under infected conditions, myeloid cells are arrested in an immature state and possess a potential capacity to suppress immune cell responses by creating a suppressive environment; such cells are termed MDSCs. MDSCs have gained much attention due to their roles in immunosuppression and in the promotion of angiogenesis [33] and metastasis [34]. MDSCs exert immunosuppressive effects depending on the expanding subtype, the disease stage and the site where immunosuppression is occurring [35], and their mechanism may change with the disease progression or the affected organ. M-MDSCs suppress T-cell function *via* both antigen-specific and nonspecific mechanisms by producing high amounts of arginase 1, NO and immunosuppressive cytokines [11, 12]. Under infection conditions, the capacity of MDSCs to differentiate into macrophages and dendritic cells is inhibited. MDSCs are more immunosuppressive than their counterparts when assessed on a per cell basis [13–15]. The mechanism associated with MDSC regulation is becoming accepted as another means of regulating immune responses, and MDSCs are potential therapeutic targets in multiple infectious and inflammatory diseases. While the underlying molecular mechanisms of MDSCs and their sub-populations remain unclear, epigenetic modification of MDSC function could provide some molecular evidence concerning MDSC accumulation and immunosuppression. Studies have confirmed that a number of lncRNAs are crucial in biological processes, including regulating gene expression, cell development and metabolism, which are disordered in disease [18, 19, 36]. A global lncRNA expression profile specific to a functional MDSC population is not known. In the present study, we selected splenic M-MDSCs for lncRNA microarray analysis based on previous studies [4, 13, 15] to investigate and compare the lncRNA expression profile of M-MDSCs and to discover the role and significance of lncRNAs in M-MDSCs, which indicated that lncRNA is a mediator that recruits MDSCs in Eg-psc-infected models.

We compared the lncRNA expression profiles between splenic M-MDSCs from Eg-psc-infected mice and normal mice to identify the lncRNAs that may be important in M-MDSC differentiation and function. Compared with the normal mice, 582 lncRNAs were upregulated and 67 lncRNAs were downregulated, and 28 upregulated mRNAs and 1043 downregulated mRNAs were differentially expressed in the infected mice. The expression differences in the microarray detection were consistent with that of qRT-PCR detection. For further analysis of the differentially expressed lncRNAs, we constructed a co-expression network to investigate the correlation between each aberrant lncRNA and their target mRNAs. GO analysis and KEGG pathway analysis were carried out to investigate the biological functions of lncRNAs. The GO enrichment assay showed that the differentially expressed lncRNAs were mostly enriched in positive regulation of TOR signaling in biological processes. The TOR pathway is well recognized as being related to cell proliferation and metabolism. Moreover, mammalian TOR (mTOR) is an intrinsic factor essential for M-MDSC differentiation and immunosuppressive function [37]. KEGG pathway analysis showed that the mRNAs co-expressed lncRNAs were mainly involved in regulation of the actin cytoskeleton, leishmaniasis, and the VEGF signaling pathway, which have been widely researched and demonstrated to be associated with MDSCs [26]. These differentially expressed lncRNAs also participate in inflammatory signaling pathways, such as the MAPK signaling pathway, tumor necrosis factor (TNF) signaling pathway, and the nuclear factor kappa B (NF-κB) signaling pathway [38, 39]. Moreover, these inflammatory pathways have been widely researched and demonstrated to be associated with MDSCs function. The vascular endothelial growth factor (VEGF) is an important molecule involved in angiogenesis. Ostrand-Rosenberg et al. [38] demonstrated that inflammation could increase MDSCs levels by protecting MDSCs from Fas-mediated apoptosis through activation of the MAPK pathway. IL-33 [40] induced arginase-1 expression and activated the NF-κB and MAPK signaling pathways, augmenting the immunosuppressive ability of MDSCs. To further study the roles of specific lncRNAs in M-MDSCs, we predicted their corresponding mRNAs through *cis*- and *trans*-targeting. We found that 288 lncRNAs were considered *cis*-regulatory lncRNAs of their sense-overlapping genes; among them, NONMMUT021591 was predicted to *cis*-regulate *Rb1*. *Rb1* expression is associated with abnormal M-MDSC differentiation. Youn et al. [28] demonstrated a novel regulatory mechanism of myeloid cells in cancer. Transcriptional silencing of the *Rb1* gene altered M-MDSC differentiation into macrophages and dendritic cells to preferential differentiation towards PMN-MDSCs. Furthermore, M-MDSCs in tumor-bearing mice could acquire the phenotypic and morphological features of PMN-MDSCs. Three hundred and seventy-two lncRNAs were predicted to interact with 60 transcription factors; several among them, namely FOSL1, YY1, IL6 and PGR, were the most enriched terms. Among these transcription factors, C/EBPβ [27] has been demonstrated to be a MDSC transcription factor, and under inflammation and infection conditions, C/EBPβ could regulate myeloid cell development and differentiation and control emergency granulopoiesis [7, 30]. Increased expression of the transcriptional regulator C/EBPβ is a characteristic biochemical feature of MDSCs. In the present study, FR015378 was predicted to be regulated by C/EBPβ and participates in the VEGF signaling pathway in KEGG analysis. Therefore, it is reasonable to propose that the aberrantly expressed lncRNAs participate in parasitic infection induction of M-MDSCs by acting with their correlated mRNAs and transcription factors.

## Conclusions

LncRNAs are critical in modulating the immune microenvironment and MDSCs; our findings provide a new understanding of M-MDSCs, and the data we present could guide the exploration of lncRNA-mediated immunosuppression in long-term parasitic infection. Although the sensitivity and specificity of lncRNA biomarkers in M-MDSCs should be further investigated, the functional lncRNAs can be explored as potential biomarkers or novel treatment strategies for immunoregulation in related diseases. Understanding the underlying mechanisms and functions of these immunosuppressive cell populations will pave the way for new parasite vaccine strategies.

## Additional files

Additional file 1: **Table S1.** Primers used in qPCR detection of selected lncRNAs. (XLSX 10 kb)

Additional file 2: **Table S2.** Significantly and differentially expressed lncRNAs in M-MDSCs. (XLSX 49 kb)

Additional file 3: **Table S3.** Significantly and differentially expressed mRNAs in M-MDSCs. (XLSX 83 kb)

Additional file 4: **Figure S1.** The lncRNA NONMMUT021591 was predicted to *cis*-regulate the protein-coding gene *Rb1*. Red dots, genomic location of lncRNAs; blue dots, the corresponding genes; rho value, correlation coefficient. (TIF 13 kb)

## Abbreviations

C/EBPβ: CCAAT/enhancer binding protein beta
Eg-psc: *Echinococcus granulosus* protoscoleces
GO: Gene Ontology
KEGG: Kyoto Encyclopedia of Genes and Genomes
lncRNA: long non-coding RNA
MAPK: mitogen-activated protein kinase
MDSCs: myeloid-derived suppressor cells
M-MDSCs: monocytic myeloid-derived suppressor cells
mTOR: mammalian TOR
NF-κB: nuclear factor kappa B
NO: nitric oxide
PMN-MDSCs: polymorphonuclear myeloid-derived suppressor cells
TNF: tumor necrosis factor
TOR: target of rapamycin
VEGF: vascular endothelial growth factor
